# Assessment of total mercury (Hg) in soil, sediment, and tilapia fish (*Oreochromis niloticus*) and health risk assessment among residents of Kitwe mining area, Zambia

**DOI:** 10.1007/s11356-025-36506-0

**Published:** 2025-05-15

**Authors:** Musonda Chisanga, Ethel M’kandawire, Kennedy Choongo, Gerald Kalunga, John Yabe

**Affiliations:** 1 Department of Para-Clinical Studies, School of Veterinary Medicine, The University of Zambia, P.O Box 32379, Lusaka, Zambia; 2 Department of Disease Control, School of Veterinary Medicine, The University of Zambia, P.O 32379, Lusaka, Zambia; 3 Department of Biomedical Sciences, School of Veterinary Medicine, The University of Zambia, P.O Box 32379, Lusaka, Zambia; 4 Department of Soil and Herbage Chemistry, Mount Makulu Central Research, Zambia Agriculture Research Institute, Private Bag 7, Lusaka, Zambia; 5 Department of Para-Clinical, School of Veterinary Medicine, University of Namibia, Windhoek, Namibia

**Keywords:** Mining impacts, Mercury pollution, Exposure assessment

## Abstract

Mercury (Hg) is a heavy metal of global concern because of its persistence in the environment and its ability to bioaccumulate and biomagnify in ecosystems. Despite evidence of extensive environmental pollution in the Copperbelt Province, few studies have investigated Hg contamination in the Kafue River and its tributaries in Kitwe District, Zambia. Total Hg concentrations were determined in soil, sediments, and tilapia by inductively coupled plasma mass spectrometer (ICP-MS) from the mining areas and non-mining areas. There were significant differences in the population means for soil samples (Mean _(mining)_ = 1.066, Mean _(non-mining_) = 0.041, *p* $\le 0.05$) and sediment samples (Mean _(mining)_ = 1.304, Mean _(non-mining)_ = 0.034), *p* $\le 0.05$) between mining and non-mining areas. There were also statistically significant differences in the population means for fish samples (Mean _(mining)_ = 0.015, Mean _(non-mining)_ = 0.007, *p* $\le 0.05$) between mining and non-mining areas. The levels of Hg in the soil and sediments from the mining area were higher than the United States Environmental Protection Agency (USEPA) reference values of 0.3 mg/kg and 0.2 mg/kg, respectively. There was a weak positive correlation between the size of the fish (length) and Hg accumulation in the Kitwe mining area (*r* = 0.232, *P* = 0.1166). The observed correlation between Hg accumulation and length of fish was not statistically significant (*P* > 0.05). The EDI from the consumption of fish from the mining area was below the USEPA and WHO/FAO maximum tolerable daily intake of 0.1 µg/kg/day and 0.23 µg/kg, respectively. The THQ < 1 was also reported in the current study, suggesting that the exposure level may not cause adverse health effects during a lifetime in the human population. Although the EDI and THQ < 1 in the current study were below the USEPA and WHO/FAO maximum tolerable limit, the presence of Hg in fish in this area must be monitored due to its ability to bioaccumulate in large and predatory fish. The lower EDI value reported in the current study might be attributed to the smaller size of the tilapia fish specimens, resulting in low bioaccumulation of Hg. Since the Hg levels in sediments were above the USEPA limit, we recommend further studies on the bioavailability of Hg in humans and other fish species in the region, particularly carnivorous fish, due to Hg biomagnification to offer a clearer perspective on the environmental and health impacts.

## Background

Mercury (Hg) is a heavy metal that is found naturally in the environment in four forms, namely: elemental mercury (Hg^0^), mercurous mercury (Hg_2_^++^), mercuric mercury (Hg^++^), and methyl mercury (CH_3_Hg^+^). Mercury accumulation in biota occurs after Hg is transformed into its organic form, methyl Hg, usually in sediments. Environmental Hg contamination can be caused by many anthropogenic activities, such as gold mining and the non-ferrous metal smelting of copper (Cu), zinc (Zn), cobalt (Co), and lead (Pb) ores. Elevated Hg concentrations have been reported from smelter dust, copper concentrate crushes, and boiler dust from the mining and smelting operations in Copperbelt Province (Kříbek et al. 2010). The main global source of anthropogenic Hg emissions is artisanal small-scale gold mining (ASGM), which uses Hg to extract gold from its ore (Futsaeter and Wilson 2013). Almost all the Hg used during the extraction process is lost to the environment, at an estimated 1400 metric tonnes per year globally (Futsaeter and Wilson 2013). The Hg released in the environment during mining increases the concentration in the atmosphere and water bodies, where it is converted to methyl Hg (Spiegel and Veiga 2005). The mining and smelting of non-ferrous metal ores such as copper are also some of the most important sources of Hg pollution in numerous countries (Ettler et al. 2007; Li et al. 2013). According to the studies by Pirrone et al (2010), ore mining and processing is responsible for about 13% of global Hg emissions.

Mercury in all its forms is a neurotoxicant that bioconcentrates to cause Minamata disease, which is characterized by permanent brain damage, central nervous system disorders, memory loss, liver damage, kidney damage, and loss of sensation (Zafar et al. 2024). Minamata disease was first reported in Minamata Bay in Japan in 1956 (Harada 1995). After the Minamata incident, Hg poisoning has been reported in other parts of the world. The severity of the health effects of Hg is dependent on the level of exposure. Methyl Hg is of the greatest concern in terms of human and animal exposure due to its ability to bioaccumulate in aquatic ecosystems and biomagnify in the food chain (Morel et al. 1998). The United Nations Environment Program (UNEP) report (2013) shows that fish-eating birds and pregnant mothers are equally vulnerable to methyl Hg exposure. However, the mechanisms and pathways of exposure may differ. Fish-eating birds are exposed to methyl Hg through their diet, primarily consisting of predatory fish with high Hg accumulation (Zamani-Ahmadmahmoodi et al. 2009). In humans, pregnant mothers, infants, and children are most at risk of methyl Hg because of its ability to bioaccumulate in the placenta, foetal tissue, and amniotic fluids (Björkman et al. 2007). Methyl Hg is easily absorbed into the bloodstream and can readily diffuse through the placental and blood–brain barrier due to its organic properties (Park and Zheng 2012). Young children and foetuses are the most vulnerable to methyl Hg exposure due to the sensitivity of their nervous system development (Counter and Buchanan 2004).

Copper mining and smelting have dominated the mining industry in Zambia for more than eight decades (Sikamo et al. 2016). As such, environmental contamination has been reported in the Copperbelt Province of Zambia (M’kandawire et al. 2017). However, studies on Hg contamination in sediments and fish around the Kitwe District are rare. Despite evidence of the extensive pollution of water bodies in Africa (Yabe et al. 2010), few studies have examined the impact of Hg pollution on freshwater fish over the past decade. A study conducted by Podolský et al. (2015) showed that traces of Hg concentration were recorded in soils near the mining/smelting areas in the Copperbelt Province of Zambia and in the northern part of Namibia. Additionally, soils near the smelters and slug dumping grounds in the Copperbelt Province showed an increase in heavy metal concentrations such as Cu, Co, Pb, Zn, and Hg (Kříbek et al. 2010). The effluents from the mines in Kitwe mining areas and other industrial areas are directed towards the three main streams (including Mindolo, Kitwe, and Uchi streams), which are used as effluent channels for pollutants in Kitwe District. According to the literature, few studies have been done to investigate the contamination of Hg in soil, sediments, and fish along the Kafue River and its tributaries in the Kitwe mining area. The current study aimed to determine the levels of total Hg in soil, sediment, and fish in the Kitwe mining area in Kitwe District, Zambia. For this reason, the current study was conducted to build on the already existing knowledge of Hg accumulation in soil by extending the analysis in sediment and fish in the Kitwe mining area. The study also assessed whether the concentrations of Hg in the study area were within the recommended guidelines by the United States Environmental Protection Agency (USEPA) and the World Health Organization (WHO). The study also assessed the human health risk of residents of the Kitwe district from the consumption of fish from the Kafue River.

## Methods

### Study area and sampling locations

**Fig. 1:**
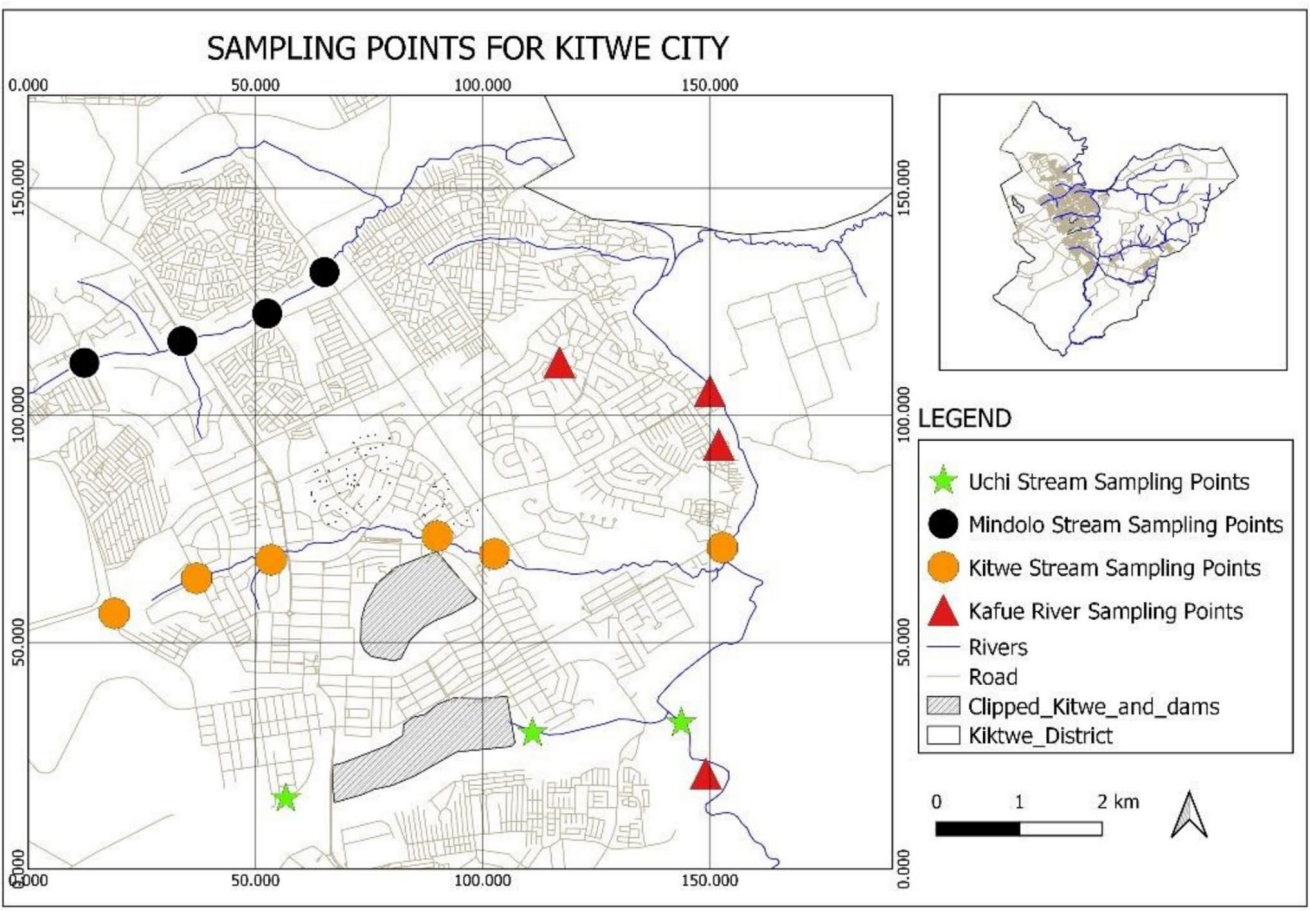
Map of Kitwe District showing sampling points along the three streams (Uchi, Kitwe, and Mindolo) connecting to the Kafue River

The study was conducted in Kitwe District (latitude − 12° 48′ 8.78″ South and longitude 28° 12′ 47.63″ East) in the Copperbelt Province of Zambia, which has a long history of copper mining as early as 1928 (Sikamo et al. 2016). Kitwe is the third largest city in terms of infrastructure development and the second largest city in terms of size (812.5 km^2^) in Zambia. The study was conducted along Kitwe streams and the Kafue River, as shown in Fig. 1. Chilubula area, which is about 45 km away from Kasama central business area in the Northern Province of Zambia and has no mining history, was also included in the study as a control site. Chilubula area is predominantly inhabited by farmers engaged in tomato and vegetable farming due to the abundance of water from the Lukupa and Luombe rivers. Figure 1 shows a map of Kitwe District and sampling points along the three streams connecting to the Kafue River.

### Sample size

The sample size for both the mining area in Kitwe (exposed) and the non-mining area (unexposed) was calculated using the Epi Info ^TM7^ (CDC, USA) stat calculator for a cross-section study design (two-sided confidence level = 95%, power 0.88%, odds ratio = 2.5). The desired sample size for all samples was *n* = 244 (mining area *n* = 96 and non-mining area *n* = 148). The mining site included soil (*n* = 32), sediments (*n* = 32), and fish (*n* = 32), and the non-mining site included soil (*n* = 50), sediments (*n* = 51), and fish (*n* = 47).

### Sample collection

The sampling activities were conducted in October 2023, during the dry season. The soil, sediment, and fish samples were collected from both the mining and non-mining areas. The collection of these samples was done according to the USEPA method 1631 for the determination of low-level Hg (USEPA 2002).

### Soil sample collection

The composite soil sampling (Ferreira et al. 2015), which consists of physical probes of grab samples being taken at randomly chosen sites throughout an entire sampling area, was adopted for the selection of soil samples in the study area. Upon the location of the sampling point for excavation, vegetation covers and stones were removed from the surface at the sampling site. The depth was delimited with the garden dibble to dig a “V”-shaped hole of depth approximately 5 cm and placed in clean polyethylene plastic. A slice of the plough layers cut was set at 20-step intervals according to the area covered within the sampling unit. About 20 soil subsamples were collected from different sites within the sampling points using a garden dibble and placed in a clean plastic bucket and a trowel. The collected soil samples were thoroughly mixed and divided into four quarters on a piece of clean paper. The two opposite quarters were discarded, and the rest of the soil was mixed again. These processes were repeated several times until 200 g of soil sample was left as a composite sample. The composite samples were labelled appropriately and stored in a clean polyethylene plastic bag (EPA SW-846). The samples were transported to Zambia Agriculture Research Institute (ZARI), Chilanga District, where analysis for total Hg was done within 28 days according to EPA holding time (EPA SW-846).

### Sediment sample collection

The sediment samples were composed of four subsamples, collected equidistantly at each of the cardinal points located radially around in a circle. At each point, four subsamples of 100 g were collected, which were then homogeneously put in a plastic bucket until a 100-g homogeneous composite sample was obtained according to the method by Caballero-Gallardo et al. (2022) with slight modifications in sample size and mass collected. The samples were packed in clean labelled polyethylene plastic bags for storage and packed in cooler boxes for transportation to ZARI, where they were kept in a refrigerator at 4°, as recommended by USEPA. The samples collected 0–7 km from the mines were categorized as upper stream and samples collected 7 km from the mines to the confluence point at the Kafue River as the lower stream.

### Fish collection

Purposive sampling of fish was adopted since the study targeted only tilapia fish (*Oreochromis niloticus*) with a predetermined average size. Thirty-two medium-sized 12–15 cm tilapia samples were collected from the sampling points along the tributaries and Kafue River using gill nets and fishing rods. Tilapia fish was used in the current study because it is the most consumed fish in Zambia, as well as being a plant and sediment eater that acts as a sink for heavy metals. Forty-seven medium-sized tilapia fish (about 12–15 cm in length) were also caught from the sampling sites in Chilubula, Kasama District. All the sampled fish were identified by popular local names (*Pale*) and scientific names. The fish were measured to standard length (cm) and weighed (g) on the scale, and a muscle tissue sample was collected and put in labelled polyethylene plastic bags and stored in a cooler box with ice packs. They were transported to ZARI Centre, Soil Chemistry Laboratory, where they were stored in a refrigerator at a temperature of 20 °C until analysis (Palacios-Torres et al. 2018).

### Sample preparation

#### Soil and sediment sample preparation

To optimize the digestion procedure, polytetrafluoroethylene (PTFE) containers were used to prevent the loss of Hg. Key parameters such as digestion time, reagent volume, and digestion temperature were optimized, where 10 mL of aqua regia (a 1:3 mixture of HCl and HNO₃) was added to 0.5 g of each soil and sediment sample. The mixture was allowed to stand overnight without heating for pre-digestion. Subsequently, it was digested at 180 °C for 2 h, with a Berghof Teflon watch glass covering the container. The Berghof Teflon watch glass was tightly closed, and an ice pack was placed on top of the Teflon glass, creating a closed system and minimizing Hg vapour loss at high temperatures. Temperature and pressure were also monitored to control excessive Hg vapour formation. After digestion, the Berghof Teflon watch glass was removed, and the sample was allowed to cool. Following this, 25 mL of 5% HCl was added, and the temperature of 180 °C was maintained for approximately 10 min. The solution was then cooled to room temperature and filtered through a syringe filter (0.45 µm) following the procedure reported by Wilson et al. (2005). The solution was carefully adjusted to a final volume of 50 mL using distilled water in a volumetric flask with 2% HNO_3_ acid.

#### Fish sample preparation

The fish samples were dried at 80 °C and homogenized using a pestle and mortar. About 0.5 g of the homogenized fish muscle was put in Ultra PTFE tubes (pre-rinsed in 7 M HNO_3_), and 5 ml of Ultrapure HNO_3_ was added. The mixture was digested at 190 °C in an UltraCLAVE (Milestone S.r.L, Italy) for 30 min. After digestion, 1 ml of analytical-grade concentrated HCl was added (to prevent loss of Hg) and diluted to 50 ml with distilled water according to a procedure reported by Simukoko et al. (2022).

### Metal analysis and quality control

The concentrations of total Hg in the soil, sediment, and fish were determined using the agilent inductively coupled plasma mass spectrometer (ICP-MS 7700, Agilent USA) at ZARI. The inductively coupled plasma multi-element standard solutions XIII (SPEX Certipur®, USA) (levels of 0, 0.5, 1, 5, 10, 20, 50, and 100 µg/L of total Hg in 1wt % HNO_3_) were used to prepare the calibration curve. The calibration curves with r^2^ > 0.999 were accepted for concentration calculation. The detection limit for Hg by ICP-MS was 0.000001 mg/L. To achieve precision and accuracy during the analysis, certified Hg reference standards (NIST SRM-2710, NIST-2709, and DORM-3) for different samples were prepared in triplicates and quintuplicates and analyzed using ICP-MS according to the guidelines. The analytical procedures were subjected to strict quality control measures. The glassware and Teflon crucibles for the analysis were pre-cleaned in 10% HNO_3_ for 15 h to minimize contamination. A concentration of 50 µg/L gold (Au) solution was spiked to all the samples to amalgamate the Hg within the samples and standards to minimize losses (Allibone et al. 1999). All samples were spiked with a concentration of rhodium (Rh) to monitor the recovery, which ranged from 100 to 101%. The standard reference materials NIST SRM-2710, NIST-2709, and DORM-3 were analyzed using the same method to check the quality assurance and method validation. The recoveries of Hg in the spiked blank and sample matrix samples ranged from 95 to 101%, respectively. For cross-contamination examination, blank samples were carried out with every 10 samples (soil, sediment, and fish) for a particular analysis. Standard samples were also analyzed with every batch of 10 samples to assess the accuracy of the analysis. The standards were prepared and used within 24–48 h to maintain the stability of Hg as guided by the USEPA method 7473.

### Estimated daily intake (EDI) through the consumption of fish

The estimated daily intake (EDI) of Hg through fish consumption depended on the following: Ci, the mean concentration of Hg in fish from the study sites; *D*, fish consumption rates; and BW, the individual fish consumer’s body weight (70 kg for adults and 18 kg for children aged 5 years). The formula below was used to calculate EDIs:

1 $$EDI=\frac{D \times \text{ Ci}}{Bw}$$

where *D* for fish 25 g/day/person and 12 g/day/person in adults and children, respectively, are the average consumption rates (Kaminski et al. 2018); Ci (mg/kg) denotes Hg concentration in fish respectively, and BW (kg) is the average body weight.

### Target hazard quotients

The human health risks of Hg exposure from consuming fish were determined based on the dimensionless target hazard quotients (THQs). The method of estimating health risk using THQs was described in the USEPA Region III risk-based concentration table (USEPA 2014). The formula for THQ is:

2 $$THQ=\frac{EFR\;x\;ED\;x\;EDI}{\mathrm{RfD}\;\mathrm x\;\mathrm AT}\times10^{-3}$$

where EFr is the exposure frequency (365 days/year); ED is the exposure duration (64 years) equivalent to life expectancy in Zambia (Shumba et al. 2024), and EDI is determined by Formula 1 above. RfD is the oral reference dose for Hg (0.1 µg/kg/day) obtained from the Integrated Risk Information System (USEPA 2014), and AT is Hg’s chemical toxicity due to the averaging lifetime exposure to Hg (365 days/year × number of exposure years, assuming 64 years).

### Statistical analysis

The concentrations of Hg levels in soil, sediment, and tilapia fish were presented in mg/kg. The statistical analyses were performed using SPSS version 20 (IBM Analytics, Armonk, NY). The distribution of data was tested for normality using the Kolmogorov–Smirnov test, and the results showed no evidence of non-normality in the data set. Therefore, the independent Student’s *t*-test was adopted for the analysis of Hg in various samples. A *p*-value of 0.05 was considered to indicate statistical significance. The Pearson correlation analysis was used to analyze the relationship between the size (weight and length) of fish and Hg accumulation in fish in the mining area. The human health risk assessments from the consumption of fish were calculated using THQs. THQ < 1 indicated unlikely chances of the exposed population experiencing adverse health effects as opposed to THQ > 1, signifying that health risks in the exposed population are significant (Barone et al. 2015).

## Results

### Quality assurance

Table 1 shows three standard reference materials for different types of samples and their recovery rates. The soil standard reference material recovery rate was 100.9%, the sediments standard reference material recovery rate was 100%, and the fish standard reference material recovery rate was 101%. The soil and sediment samples were prepared and analysed in 3 replicates per sample, and the fish samples were analysed in 5 replicates per sample to improve quality assurance.

**Table 1:** Certified and observed mercury concentrations of the standard reference materials

Standard reference material	Code on the standard	Certified	Observed	Recovery (%)
Soil (mg/kg)	NIST SRM-2710	32.6 ± 1.8	33 ± 0.61	100.9
Sediment (mg/kg)	NIST-2709	1400 ± 80	1396 ± 23	100
Fish protein (mg/kg)	DORM-3	382 ± 60	387 ± 23	101

### Mercury levels in soil, sediments, and fish (dry weight) from the mining and non-mining areas

Table 2 shows levels of Hg in soil samples from the mining and non-mining areas. The levels of Hg concentration ranged from 0.013 to 4.12 mg/kg for the mining areas and 0.012 to 0.12 mg/kg for the non-mining areas. The mean Hg concentration in the mining areas (1.06 mg/kg) was higher than the mean Hg concentration in the non-mining areas (0.041 mg/kg). The levels of Hg in the soil from the mining area were higher than the USEPA reference value (0.3 mg/kg).

**Table 2:** Descriptive statistics of Hg levels in dry weight (dw) in soil (mg/kg), sediment (mg/kg), and fish (mg/kg) in the mining and non-mining areas

Exposure	*N*	Mean	Median	Tr.Mean	Std. deviation	Std. Er. mean	Min	Max	Q1	Q2
Mining area (soil)	32	1.066	0.431	0.961	1.288	0.228	0.013	4.12	0.05	1.853
Non-mining area (soil)	50	0.041	0.036	0.039	0.0307	0.004	0.012	0.12	0.01	0.06
Mining area (sediment)	32	1.304	0.26	1.148	1.699	0.300	0.066	5.82	0.06	2.842
Non-mining area (sediment)	51	0.034	0.033	0.03	0.030	0.004	0.003	0.18	0.007	0.046
Mining area (fish)	32	0.015	0.014	0.138	0.0101	0.0017	0.00	0.045	0.007	0.021
Non-mining (fish)	47	0.007	0.008	0.006	0.0136	0.0020	0.00	0.054	0.00	0.009

Levene’s test (see Table 5 in the Appendix) for equality of variances across the soil samples in the mining and non-mining areas shows that the variances for the population from which the samples were drawn were not equal ($p \le 0.05)$. There was a statistically significant difference in the population variances for soil (*F* = 109.994, *p* $\le 0.05$). Subsequently, the *t*-test for equality of means was conducted under the “Hg equal variances not assumed” assumption for soil samples. The independent *t*-test result shows that there was a statistically significant difference in the population means for soil samples collected from mining and non-mining areas (MD = 1.025, df = 31.022, *p* $\le 0.05$).

Table 2 shows concentrations of Hg in sediment samples from the mining and non-mining areas. The levels of Hg concentration ranged from 0.066 to 5.82 mg/kg in the mining areas and 0.003 to 0.18 mg/kg in the non-mining areas, respectively. The Hg mean concentration in the mining areas (1.304 mg/kg) was higher than the Hg mean concentration in the non-mining areas (0.034 mg/kg).

Table 6 in the Appendix shows Levene’s test for equality of variances across the sediment samples in the mining and non-mining areas. The results show that the variances for the population from which the samples were drawn were not equal ($p\le 0.05)$. There was a statistically significant difference in the population variances for sediment (*F* = 126.398, *p* $\le 0.05$). Subsequently, the *t*-test for equality of means was conducted under the “Hg equal variance not assumed” assumption for sediment samples. The independent *t*-test result shows that there was a statistically significant difference in the population means for sediment samples collected from mining and non-mining areas (MD = 1.271, df = 31.012,* p* $\le 0.05$).

Table 2 shows levels of Hg in fish samples from the mining and non-mining areas. The levels of Hg concentration ranged from 0 to 0.045 mg/kg in the mining areas and 0 to 0.054 mg/kg in the non-mining areas. The mean Hg concentration in fish in the mining area (0.015 mg/kg) was higher than the mean Hg concentration in the non-mining areas (0.007 mg/kg).

Levene’s test (see Table 7 in the Appendix) for equality of variances across the tilapia fish samples in the mining and non-mining areas shows that the variances for the population from which the samples were drawn were equal ($p\ge 0.05)$. There was no statistically significant difference in the population variances for fish samples (*F* = 0.610, *p* $\ge 0.05$). Subsequently, the *t*-test for equality of means was conducted under the “equal variance assumed” assumption for fish samples. The independent *t*-test result shows that there was a statistically significant difference in the population means for the fish samples collected from mining and non-mining areas (MD = 0.0067, df = 77, *p* $\le 0.05$).

### Mercury accumulation in sediments in the upper and lower stream in the mining area

Levene’s test for homogeneity of variance shows that variances were not equal (*F* = 39.15, *P* = 0.00 < 0.05). Therefore, under equal variances not assumed, the results show that there was a statistically significant difference in Hg means in sediments between the upper and lower stream (*t* = − 2.653, df = 17.262, *P* = 0.017 < 0.05). The mean concentration of Hg in upper sediments was higher than the mean concentration of Hg in lower sediments.

### The relationship between the size of tilapia fish and mercury accumulation in the mining area

**Fig. 2:**
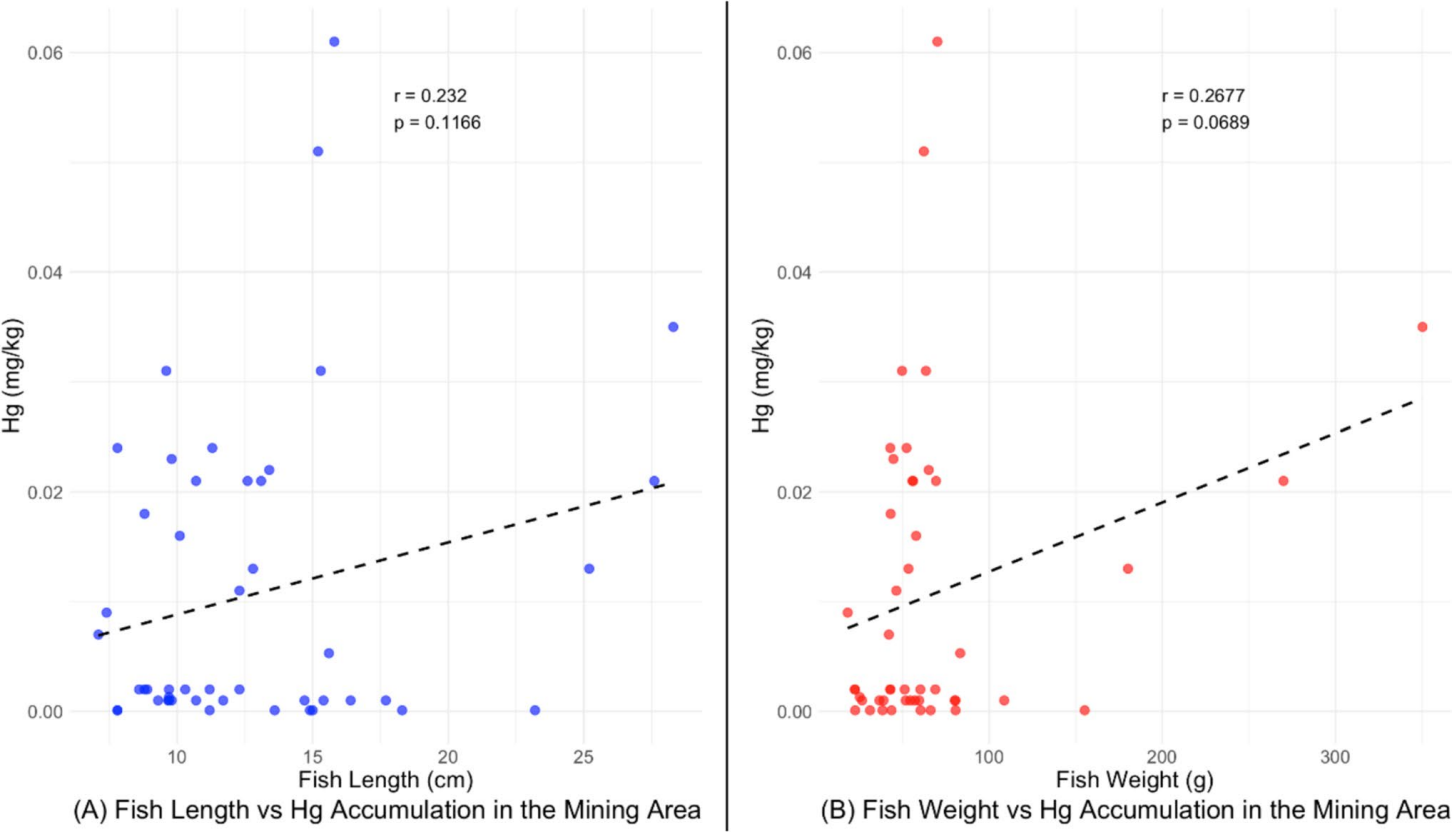
Pearson correlation between size of fish (length and weight) and mercury accumulation in Kitwe mining area

Figure 2 results show that there was a weak positive correlation between the size of the fish (length) and Hg accumulation in the Kitwe mining area (*r* = 0.232, *P* = 0.1166). The observed correlation between Hg accumulation and length of fish was not statistically significant (*P* > 0.05). The Pearson correlations between the weight of the fish and Hg accumulation results also showed a weak positive correlation *(r* = 0.267, *P* = 0.0689). The observed correlation between the weight of fish and Hg accumulation was not statistically significant (*P* > 0.05).

### Dietary intake of mercury in tilapia fish

The EDIs through the consumption of fish from the mining area were 0.005 µg/kg and 0.01 µg/kg body weight for adults and children (5 years old), respectively. The EDIs through the consumption of fish from the non-mining area were 0.003 and 0.004 µg/kg, respectively. The EDIs in all the cases were below the Food and Agriculture Organization/World Health Organization (2002) recommended standard for total Hg in fish (0.1 µg/kg).

### Health risk assessment

The THQs through the consumption of fish from the mining area were 0.05 and 0.10 for adults and children (5 years old), respectively. The THQ through the consumption of fish from the non-mining area were 0.03 and 0.04 for adults and children (5 years old), respectively. The results show that the level of exposure to Hg through the consumption of fish from both the mining and non-mining areas were within the safe limits (THQ < 1).

## Discussion

### Mercury concentration levels in soil, sediment, and fish

The present study revealed that the Hg concentration in the soil and sediments from the mining areas were higher than those from the non-mining sites. For decades, the mining industry has been recognized as one of the most important sources of environmental pollution (Plumlee and Morman 2011). These results agree with the study by Nachiyunde et al. (2013), which showed that heavy metal pollution in Zambia has strong regional differences, where areas geographically distant from the mines had low pollution concentrations. These concerns have triggered assessment efforts in heavy metal in soil and mine waste dumpsites to mitigate hazards to the ecosystem. The elevated levels of Hg in Kitwe town soils could have been due to the deposition of elemental Hg during copper metal smelting processes (Feng et al. 2009), which could have been washed off to the nearby streams.

It is known that mining and smelting of non-ferrous metal ores such as copper are some of the most important sources of Hg pollution in numerous countries (Ettler et al. 2007). Therefore, it is likely that the increase in Hg concentrations in Kitwe mining soils and sediments could be from mining activities that have been going on since the 1920 s (Sikamo et al. 2016). The levels of total Hg in soil from the mining areas were higher than the naturally global Hg background concentration value of 0.1 mg/kg recommended by USEPA (Rajaee et al. 2015). High concentrations of heavy metals (Co, Cu, Hg, Pb, and Zn) in topsoil (0–10 cm) near the Nkana mine smelter have also been reported by Kříbek et al. (2023). The average Hg levels in sediment in the current study in the Kitwe mining area were also above the USEPA recommended limit (0.2 mg/kg) of Hg in sediments and higher than the levels of Hg in sediments from the non-mining area. The sediment samples from the mining area had the highest accumulation of Hg amongst all the sample types. The total Hg level in sediments (1.30 mg/kg) was also higher than the total Hg in soil (1.07 mg/kg) and fish (0.015 mg/kg) in the Kitwe mining area. The Hg content in the aquatic bottom sediment of the reservoir is usually either equal to or 1.5 times higher than in the soils of its catchment area (Peng et al. 2018). This is because bottom sediments act as sinks and final depots of migration of some elements. These Hg levels in sediments could also be caused by mining activities and industrial wastes from metal smelting in the Kitwe urban area. The concentration of Hg in sediment samples was also higher in streams compared to the Kafue River, and this might be attributed to the shorter distance between the streams and the mining smelters and industrial areas.

The results (Table 3) show that there was a statistically significant difference in Hg means in sediments between the upper and lower stream (*P* = 0.017 < 0.05). The concentration of Hg in sediment samples collected from the upper part of the stream near the mine was significantly higher (0.82 mg/kg) than the concentration of Hg in sediment samples collected from the lower side of the stream (0.26 mg/kg). Upper sediments near the mining areas are closer to the potential Hg pollution source from the mines. Heavy metal contaminations (Hg and Pb) were observed to be elevated in areas closer to the mining area compared to non-mining areas (Ikenaka et al. 2010). Many mine drainage waters in the Kitwe mining area have low pH and carry a high concentration of metals and metalloids that settle in sediments (Kříbek et al. 2023). The low pH in mine drainage systems increases the solubility of Hg and Hg binding to other particles in upper sediments. The adsorption of Hg in bottom sediment is influenced by the lower pH of sediments (Boszke et al. 2003). Mercury can bind to particles and other organic matter, which accumulate in sediments, leading to higher concentrations in sediments closer to Hg sources (Gray et al. 2000). The elevated Hg concentration in sediments closer to the mining area may also be attributed to dust fall offs and smelter emissions into the ground, which are washed off into the streams during the rainy season and settle in sediments. Studies have reported high Hg concentrations from smelter dust, dust from slag crushes, and electrostatic precipitator dust from the mining and smelting operations in the Copperbelt Province (Kříbek et al. 2010; Podolský et al. 2015). The lower Hg levels downstream might also be influenced by dilution, especially in the rainy season. The volume of water increases as it flows downstream, diluting Hg concentrations.

**Table 3:** Levels of total Mercury in sediments in dry weight (dw) from the upper and lower parts of the Kitwe stream

Stream part	*N*	Mean	Std. deviation	Std. mean
Hg upper	16	0.82	1.02	0.43
Hg lower	16	0.26	0.37	0.097

In a study near a gold mine in Congo DR, a mean Hg concentration of 13.5 mg/kg was reported in Kimbi River sediments (Pascal et al. 2020), which was more than 13 times higher than in the current study. These findings provide evidence that suggests that a large source of Hg to the environment is ASGM, which releases 1400 tons of Hg annually into the environment globally (Futsaeter and Wilson 2013). This may be due to the use of large quantities of Hg to separate gold from sediments during gold extraction from its ore by ASGM compared with copper smelting that does not require the use of Hg. The sources of Hg in soil and sediment in Kitwe, Zambia, might be due to the increasing non-ferrous metal smelting because of the pyrometallurgical treatment of Cu, Pb, and Zn concentrates in the Kitwe mining and industrial area. Direct emissions from mining and ore processing facilities are often accompanied by toxic dust emissions and effluents from waste disposal sites of floatation tailing, waste rock, and metallurgical slags. The mining and smelting of non-ferrous metal ores such as Cu are some of the most important sources of Hg pollution in numerous countries (Ettler et al. 2007; Li et al. 2013). These findings agree with the observations reported by M’kandawire et al. (2017) on high heavy metal pollution in the Copperbelt Province.

The levels of Hg in tilapia reported in the current study were lower than the reported Hg levels by Kola et al. (2019), who reported higher total Hg levels in Nile tilapia (*Oreochromis niloticus*) in rivers near a gold mine in Kenya above the 0.5 mg/kg recommended by USEPA. This variation could be attributed to the direct use of Hg during gold mining that biomagnifies after the methylation process and contaminates the feeding ecology of the fish. Methyl Hg accumulates in the environment and biomagnifies in the food chain, posing high risks to human health through the consumption of contaminated fish (Gibb and O’Leary 2014).

The increase in Hg concentration in fish in the Kitwe mining area could be attributed to the increase in effluents from the mining and smelting areas deposited into the nearby streams flowing into the Kafue River. Although the levels of Hg in sediments were higher than the USEPA limit, the Hg bioavailable for transfer from sediment to tilapia fish was low. This could be due to the habits of fish species, dietary structure, trophic level, and size of the fish in the current study, as Hg accumulation in fish is positively related to the fish’s size (Wiener et al. 2003). The poor correlation (Fig. 2) between the length of fish and Hg accumulation observed in streams and the Kafue River in the Kitwe mining area might be attributed to low bioaccumulation in fish due to smaller fish size and age. Mercury loading in fish may be influenced by size, age, diet, and broader environmental conditions such as temperature and level of contamination (Verdouw et al. 2011). Most of the fish sizes were smaller in the mining area, especially in the streams, and this may be attributed to the smaller water volume, which can limit the amount of food and habitat available for fish. The mining effluents released in the streams also contributed to the destruction of the fish habitat and breeding grounds. In Kitwe, heavy metal pollution originates from the Uchi mine tailings, Nkana smelter, and slag dump sites in the town’s industrial area into aquatic systems through leaching, precipitation, and overflow (Sracek et al. 2012). Many mine drainage waters have low pH and carry high concentrations of metals and metalloids, altering aquatic ecosystems (Kříbek et al. 2023). High levels of pollution in streams have also not deterred locals from overfishing, reducing the fish population and limiting the fish’s opportunities to grow. These factors could have contributed to the lower fish population and fish size in the area, affecting the bioaccumulation of Hg in tilapia in the current study. Oppong et al. (2010) also observed a poor correlation between Hg concentration and fresh weight and size of *Tilapia multifasciata*. However, there was a good correlation between Hg concentration and fresh weight (*r* = 0.803) and length (*r* = 0.845) for *Tilapia zilli*. The results reported by Oppong et al. (2010) also suggest that Hg accumulation in tilapia fish is associated with the species of tilapia. Verdouw et al. (2011) also reported that the age and size of fish significantly influence Hg accumulation in brown trout (*Salmo trutta*). These findings are not in agreement with our findings in the current study. These variations may be due to the large size and age of brown trout; hence, bioaccumulation occurs in large fish. Brown trout is also generally considered to be at a higher trophic level than tilapia (*Oreochromis*), hence higher bioaccumulation of Hg than tilapia. Simukoko et al. (2022) reported a higher concentration of Hg in wild tilapia than in farmed tilapia in Lake Kariba. The variations in the levels of Hg in wild tilapia and farmed tilapia were attributed to the size and age of the fish. Wild tilapia can live up to 9 years compared to farmed tilapia, which is harvested within 6 months. As a result, wild tilapia can accumulate Hg with a long biological half-life compared to farmed tilapia. The Hg levels reported in wild tilapia in Lake Kariba are higher than the Hg levels reported in wild tilapia fish in the Kafue River in the current study. These variations may be attributed to the longer water-residence time in Lake Kariba, which allows Hg to accumulate and biomagnify in the food chain. Unlike the lake, the Kafue River has faster water flow, reducing the residence time of Hg and limiting its bioaccumulation in fish (Drevnick et al. 2016). The reported higher Hg in wild tilapia from Lake Kariba is also attributed to the larger size of the fish and age, resulting in higher biomagnification compared to the tilapia in the current study. The reported low concentration in fish in the current study could also be attributed to the diet patterns of the tilapia fish in the area, as higher Hg levels are found in piscivorous fish than omnivorous fish and non-piscivorous fish (Ouédraogom and Amyot 2013). The higher concentrations of Hg reported in predatory fish recorded in other studies (Voegborlo and Adimado 2010; Ouédraogom and Amyot 2013) might be due to the biomagnification of Hg in their bodies since they are at the top of the food chain. The low bioaccumulation levels of Hg observed in the current study could be attributed to the use of smaller non-predatory species of fish. These levels were below the FAO/WHO-recommended limit of 0.5 mg/kg in fish, indicating minimal risks associated with the consumption of fish in the study areas.

### Assessment of potential public health risks: EDI and estimated THQ

Mercury that bioaccumulates in fish and is ingested by consumers eventually accumulates in various tissues and organs of the human body, leading to serious health implications (Khanam et al. 2020). Therefore, the human health risk assessment model was used to compare the average daily intake of Hg in fish with the daily allowable intake of Hg (0.1 µg/kg) by USEPA (2018). In the current study, the EDIs for both adults and children from the consumption of fish from the mining area were below the USEPA (2018) value of 0.1 µg/kg/day and the Food and Agriculture Organization/World Health Organization (2002) value of 0.23 µg/kg, suggesting that the exposure level may not cause adverse health effect, particularly to vulnerable populations such as children and pregnant women. The estimated weekly intake (EWI) of 0.035 µg/kg from the calculated EDI for adults (Table 4) is also less than the provisional tolerable weekly intake (PTWI) of 4 µg/kg (EFSA Panel on Contaminants in the Food Chain 2012). This indicates that the fish can be consumed regularly without any significant health risks. The smaller EDI value might be attributed to the smaller size of the tilapia fish in the current study. Studies have shown that the bioaccumulation of Hg is dependent on the age, size, and species of fish (Wiener et al. 2003). Tilapia is an omnivorous fish that mainly feeds on algae and phytoplankton, microscopic plant-like organisms, and lower-level trophic levels (Lu et al. 2006). Tilapias have a shorter food chain, which reduces the opportunity for Hg bioaccumulation, and have faster Hg elimination rates due to higher metabolic rates. Although the EDIs in the current study were below the USEPA and WHO/FAO maximum tolerable limit, the presence of Hg in fish in this area must be monitored due to its ability to biomagnify in large and predatory fish. Mercury can be harmful even at a low concentration due to its toxicity and ability to bioaccumulate in the ecosystem (Sharma et al. 2015). Studies have also shown that bioaccumulation occurs in higher trophic guilds and larger piscivore fish (Voegborlo and Adimado 2010; Ouédraogom and Amyot (2013). Piscivore fish, especially apex predators, tend to have higher bioaccumulation of Hg due to a longer food chain and slower Hg elimination rates. Therefore, future investigations must emphasize the assessment of Hg in large-size predatory fish on top of the food chain, such as African tigerfish (*Hydrocynus vittatus*) in the mining area.

**Table 4:** Table of EDI and THQs

Sample (*n* = 79)	Concentration of Hg (mg/kg) ± SD	Estimated daily intake (EDI) in µg/kg/bw/day		Target hazard quotient (THQ)	
		Adults (64 years)	Children (5 years)	Adults (64 years)	Children (5 years)
Mining area (*n* = 32)	0.015 ± 0.0101	0.005	0.01	0.05	0.10
Non-mining area (*n* = 47)	0.007 ± 0.0136	0.003	0.004	0.03	0.04
Maximum tolerable daily intake (MTDI)		0.1^a^			
		0.23^b^			

^a^USEPA (2018)

^b^Food and Agriculture Organization/World Health Organization (WHO/FAO) (2002)

The THQ < 1 reported in the current study for both adults and children signifies that the level of exposure is lower than the reference dose, which assumes that daily exposure at this level is not likely to cause any negative health effects during a lifetime in the human population. These results were similar to the THQ < 1 reported by Simukoko et al. (2022) in their Hg assessment of wild and farmed tilapia in Lake Kariba, Zambia. While the THQ < 1 indicates that fish is generally safe to eat, caution is still advised for sensitive populations, such as pregnant women and young children, due to potential long-term health effects. The residents from the study area may be exposed to higher dietary Hg concentrations through the consumption of locally grown vegetables in the study area. Studies have shown that heavy metals easily accumulate in the edible part of the leafy vegetables in plants that are grown near mineral processing areas (Mapanda et al. 2005; Muchuweti et al. 2006). Such diets could directly increase the EDIs of Hg and consecutively increase THQs from insignificant levels to significant levels. Studies of health risk assessment near gold mining areas have shown evidence of high THQ due to the direct use of Hg during gold processing by ASGM. Mercury assessment in different species of fish in the Teta River near gold mining fields in Colombia shows higher THQ > 1 values (Caballero-Gallardo et al. 2022). Due to the risk associated with Hg contamination in gold mining, it is essential to implement proper mitigation strategies, such as soil testing and irrigation management, in the National Action Plan for ASGM in Zambia.

## Conclusion

The result of our study has revealed that anthropogenic activities such as mining and non-ferrous metal smelting activities in the Kitwe urban area have contributed to the increase in Hg levels in soil and sediments. The Hg levels in the Kitwe mining area were significantly higher than in Kasama, a non-mining area. The levels of Hg in the soil and sediments from the mining area were higher than the USEPA reference values of 0.3 mg/kg and 0.2 mg/kg, respectively. The EDI from the consumption of fish from the mining area was below the USEPA and WHO/FAO maximum tolerable daily intake of 0.1 µg/kg/day and 0.23 µg/kg, respectively. The THQ > 1 was also reported in the current study, suggesting that the exposure level may not cause adverse health effects during a lifetime in the human population. Although the EDI and THQ < 1 in the current study were below the USEPA and WHO/FAO maximum tolerable limit, the presence of Hg in fish in the study area must be monitored due to its ability to bioaccumulate in large and predatory fish. The lower EDI value reported in the current study might be attributed to the smaller size of the tilapia fish specimens, resulting in low bioaccumulation of Hg. Given that the Hg levels in sediments were above the USEPA limit, we recommend further studies on the bioavailability of Hg in humans and other fish species in the region, particularly carnivorous fish, due to Hg biomagnification to offer a clearer perspective on the environmental and health impacts. While our study has some limitations, it provides a baseline for future studies in Hg exposure and health risk assessment, especially in developing the guidelines for the rapidly developing gold mining sector in Zambia.

## Data Availability

All the data included in the current study are available on request.

## Appendix

See Tables

**Table 5:** Levene’s test for equality of variances across the soil samples in the mining and non-mining areas

								95% confidence interval of the difference	
	F	Sig	T	df	Sig (2-tailed test	Mean difference	Std. error difference	Lower	Upper
Hg Equal variances assumed	109.994	0.000	5.647	80	0.000	1.025	0.182	0.66	1.386
Hg equal variances not assumed			4.503	31.022	0.00	1.025	0.228	0.561	1.489

^5^,

**Table 6:** Levene’s test for equality of variances across the sediment samples

								95% confidence interval of the difference	
	F	Sig	T	df	Sig (2-tailed test	Mean difference	Std. error difference	Lower	Upper
Hg Equal variances assumed	126.398	0.000	5.358	81	0.000	1.271	0.237	0.799	1.742
Hg equal variances not assumed			4.229	31.012	0.00	1.271	0.300	0.658	1.883

^6^ and

**Table 7:** Levene’s test for equality of variances across the tilapia samples

								95% confidence interval of the difference	
	F	Sig	T	Df	Sig (2-tailed test	Mean difference	Std. error difference	Lower	Upper
Hg Equal variances assumed	0.610	0.437	2.363	77	0.021	0.0067	0.0028	0.001	0.0122
Hg equal variances not assumed			2.499	76.407	0.015	0.0067	0.0027	0.001	0.0119

^7^
